# Pacing and Positioning Strategies During an Elite Fixed-Gear Cycling Criterium

**DOI:** 10.3389/fspor.2020.586568

**Published:** 2020-10-08

**Authors:** Nicolas Babault, Christos Paizis, Mary Trimble, David A. Trimble, Carole Cometti

**Affiliations:** ^1^Center for Performance Expertise, CAPS, U1093 INSERM, University of Bourgogne-Franche-Comté, Faculty of Sport Sciences, Dijon, France; ^2^Trimble Racing Inc., New York, NY, United States

**Keywords:** TACTICS, sex, cycling performance, rank, LAP

## Abstract

Fixed-gear cycling performance during criteriums predominantly involves the aerobic system. Whether pacing is another important factor for performance is unknown. The purpose of the present study was to explore pacing and/or positioning strategies of fixed-gear riders during criteriums. Race results of an international fixed-gear criterium were analyzed (20 laps for women and 28 laps for men; laps = 1,270 m). Statistics were conducted on individuals lap time and positioning during the finals. Race pattern in women (*n* = 35) and men (*n* = 53) revealed that the faster laps (*P* < 0.05) were in the middle and at the end of the race and the slower laps (*P* < 0.05) were at the end of the race (laps 17–18 for women and lap 26 for men). The final ranking was significantly correlated with the mean race position (Kendall's tau = 0.664 and 0.689 for women and men, respectively). A coefficient of variation >50% revealed an important positioning variability. The best riders are mostly amongst the first during the race. However, the others exhibited larger mean position variations during the first half of the race. Our results demonstrated variable pacing strategies during fixed-gear criteriums. Although some riders had economical drafting strategies during the first half of the race, riding placed ahead during the whole race seemed to be an essential performance factor.

## Introduction

Fixed-gear cycling consists of riding track bicycles outdoors generally on roads, streets or circuits. Bicycles are brakeless, with only one gear and no free wheel. Without any official instances, numerous competitions are organized worldwide. One competition format is the criterium. It is generally performed in short closed circuits (generally <2 km) with numerous turns, accelerations, and decelerations. Competitions are usually composed of different steps such as qualifications (one lap), heats (<20 min), and finals (>40 min).

To the authors' knowledge, only one study was conducted and explored fixed-gear performance determinants during criteriums using race results and laboratory evaluations (Babault et al., 2018). The main results revealed that fixed-gear cycling performance is predominantly aerobic. Although this type of cycling involves some specific skills attributed to frequent active decelerations and re-accelerations during direction changes, strength is not a key factor. Authors also indicated that fixed-gear cycling during criteriums is similar to cyclo-cross or mountain bike cycling because of some technical skills similarities.

Fixed-gear criteriums are strategic events which suggests that pacing or positioning could be important factors contributing to performance. Numerous studies have been performed during cycling (Faria et al., 2005; Davies et al., 2016). To date, no data exists for fixed-gear bike events and very few studies have explored similar cycling events in short closed circuits such as cyclo-cross (Bossi et al., 2018) or mountain bike (Martin et al., 2012; Granier et al., 2018; Moss et al., 2019).

In mountain bike cycling, pacing has been shown to be related to performance, age, sex, and race experience (Moss et al., 2019). Even or positive pacing strategies are generally observed in official races or laboratory simulated events (Martin et al., 2012; Bossi et al., 2018; Moss et al., 2019). In addition, fast starts are often observed (Bossi et al., 2018; Granier et al., 2018; Viana et al., 2018) and large within-laps variability has been observed partly as a result of topography (Martin et al., 2012; Moss et al., 2019).

However, because fixed-gear criteriums are generally flat, it is difficult to extrapolate conclusions from studies conducted in mountain bike cycling or cyclo-cross. Therefore, the aim of the present study was to explore pacing and/or positioning strategies during a top-level fixed-gear criterium to verify whether they are of paramount importance for final performance. In addition, men and women results were considered to determine possible sex specificities. We hypothesized that variable pacing strategies will be observed and that being placed ahead will be an efficient positioning strategy. Due to the lack of data available for fixed-gear cycling, the results from the present study will help practitioners to optimize training sessions and stimuli.

## Materials and Methods

### Participants

The present study involved the analysis of publicly available results of a fixed-gear criterium. Data were retrieved from direct contact with the race direction. The study was conducted according to the declaration of Helsinki and approved by the local institutional review board with a waiver for informed consent of the participants due to publicly available dataset.

### Fixed-Gear Competition

**Figure 1:**
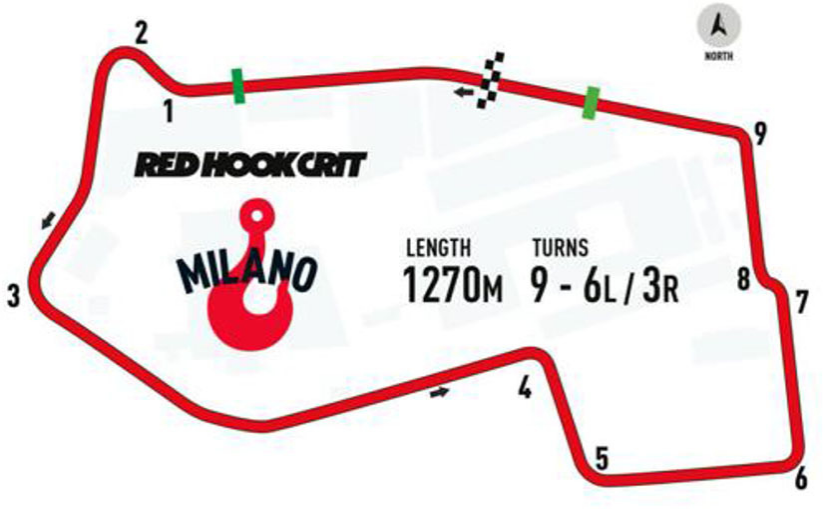
Trace of the race during the Red Hook Criterium in Milano. The direction of the race is given by the arrow. Turns are presented as numbers. The lap length, total number of turns and direction of turns (L, left; R, right) are also presented. The finishing line is presented in white and black as well as the geographical north (arrow in the gray circle).

The race used for this investigation took place in Milano (Italy) October the 6th 2018 and was a part of the Red Hook Criterium international championship. The circuit (Figure 1) was 1,270 m long, flat, with nine turns. This event took place in a single day. First, riders competed heats. For women, it consisted of two heats with eight laps and all riders were qualified to the final. For men, it was four heats of 10 laps. The first 20 riders were qualified for the final. The other riders participated in a “last chance race” that consisted of eight laps with four distinct races. These “last chance races” were performed with about 1 h after heats. Only the first five of this second race were qualified for the final.

For women, the final race was performed 4 h after heats. For men, the final was performed 6 h after heats or 3 h after the “last chance race.” The particularity of this event is that finals are competed during the night with public and additional race lightings. The position during the final was dependent on the performance during heats. Women had to ride 20 laps while men had 28 laps. Because races were performed in a closed-loop circuit, riders could be disqualified during finals. Riders were stopped just before being lapped by the head of the race.

### Statistical Analysis

From the results of this event (women and men final), we obtained the lap times (in s) and lap positioning. Women and men performances were tested separately. Data in the text are given as mean values ± standard deviation (SD). The variability was tested for lap time and position variation (position range of each rider position) using coefficients of variation (%). Statistical analyses were conducted using Statistica v8.0 (StatSoft Inc., Tulsa, USA). *P* < 0.05 was set as the level of statistical significance for all tests.

Because sphericity and normal distribution of the data were verified by using Mauchly and Shapiro-Wilk tests, parametric statistics were used for lap time. A one-way analysis of variances (ANOVA) was used to test differences between laps. The main factor (lap number) was used as a repeated measure. A two-way ANOVA was also conducted while grouping laps into four distinct parts (0–25, 25–50, 50–75, and 75–100% begin from the first lap to 25% of the race, 25–50, 50–75, and 75% to the end of the race, respectively) and sorting riders in quartiles as a function of the final position (quartile 1 being the 25% first riders at race end). In case of significant main effects or interactions, Bonferroni pairwise comparisons were conducted. Partial eta squared (partial η^2^) was calculated with values of 0.01, 0.06, and above 0.14 representing small, medium, and large differences, respectively (Cohen, 2013).

The normality was not observed for riders' position. Non-parametric tests were therefore conducted. Riders' position was first tested using Kendall's tau correlation. It permitted to determine the degree of association between the final positioning and the mean positioning during the whole race or race parts. Wilcoxon and Kruskal-Wallis followed by pairwise Bonferroni adjustments were secondly used to compare differences in riders' position as a function of the race part and riders' quartile, respectively. Position variation (calculated as the position range of each rider during the whole race or race parts; range being defined as the difference between the best and worst position) was similarly tested.

## Results

### General Observations

Three hundred and sixty-two riders (70 women and 292 men) participated in this event. After heats, 66 women were at the start of the final and 94 men. Because, riders were stopped just before being lapped by the head of the race, only 35 women and 53 men (finishers) were considered during statistics. The total race duration was 2264.6 and 2840.4 s for the winner of the women and men race, respectively. At the end of the race, the first half of the riders was separated by only 11.2 s for women and 16.3 s for men.

### Lap Time

**Figure 2:**
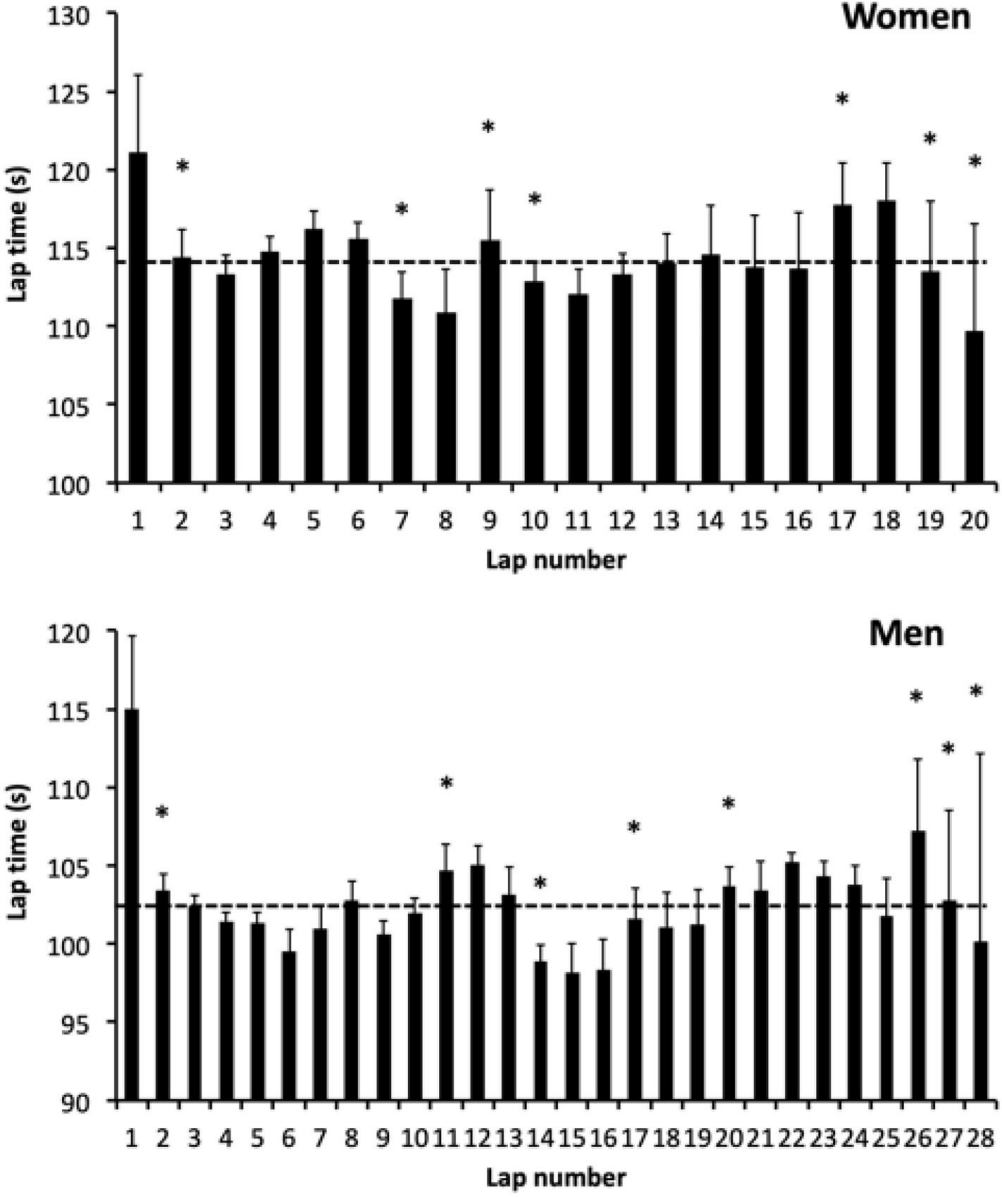
Lap time during the race for women (upper panel) and men (lower panel) riders. The horizontal dashed lines represent the race mean lap time. Differences with the preceding lap are shown (*P < 0.05). Values are means ± SD.

The mean lap time was 114.3 ± 3.9 s and 102.6 ± 4.4 s for women and men, respectively (Figure 2). Lap time showed very low variability as attested by low coefficients of variation (3.4 and 4.3% for women and men, respectively). The one-way ANOVA revealed a lap time effect during the women race (*F* = 31.4, *P* < 0.001, Partial η^2^ = 0.999, power = 1.00) and men race (*F* = 59.5, *P* < 0.001, Partial η^2^ = 0.999, power = 1.00). Women exhibited significant accelerations (lap faster than the immediately preceding) at lap 2, 7, 10, and 19. Significant decelerations (lap slower than the immediately preceding) were observed at lap 9 and 17. The fastest performances were observed for laps 7, 8, and 20. Except for the first lap, the slowest laps were observed at the end of the race (laps 17 and 18). For men, significant accelerations were obtained at lap 2, 14 and 27, 28. Significant decelerations were observed at lap 11, 17, 20, and 26. The fastest laps were laps 14–16 and 28. Except for the first lap, the slowest lap was observed at the end of the race (lap 26).

When grouping laps and sorting riders, interactions were obtained for women (*F* = 20.4, *P* < 0.001, Partial η^2^ = 0.664, power = 1.00) and men (*F* = 20.0, *P* < 0.001, Partial η^2^ = 0.551, power = 1.00, Table 1). For women and whatever the quartile, 0–25% was always the slowest part (*P* < 0.05). Seventy-five to hundred percentage was the fastest part of the race (*P* < 0.05) for Q1 (best riders) while it was the slowest (*P* < 0.001) for Q4. Q4 was slower than the others during the second half the race (50–75 and 75–100%, *P* < 0.05). For men, the first half of the race was slower than the second half (*P* < 0.05) for Q1. Q2 and Q3 exhibited the fastest laps between 50–75% of the race (*P* < 0.05) and the slowest laps between 0–25% (*P* < 0.05). For Q4, the fastest part was between 25–50 and 50–75% of the race and the slowest part was between 0–25 and 75–100%. When comparing quartiles, no difference was observed between Q1 and Q2 or between Q3 and Q4. Q1 was faster than Q3 for 75–100% (*P* < 0.001) and faster than Q4 for 0–25, 50–75, and 75–100% (*P* < 0.05). Q2 was faster than Q3 and Q4 for 50–75 and 75–100% (*P* < 0.05).

**Table 1:** Mean lap time (expressed in s) during the race for women and men.

**Sex**	**Race part**	**Q1**	**Q2**	**Q3**	**Q4**
Women	0–25%	115.4 ± 0.3^*^	115.9 ± 0.3^*^	116.1 ± 0.3^*^	116.2 ± 0.3
		(115.0; 115.8)	(115.5; 116.4)	(115.7; 116.6)	(115.7; 116.6)
	25–50%	112.7 ± 0.2^$^	113.1 ± 0.2	113.5 ± 0.2	113.7 ± 0.2^$^
		(112.5; 112.9)	(112.9; 113.3)	(113.3; 113.7)	(113.5; 114.0)
	50–75%	113.4 ± 0.5^$^	112.6 ± 0.5	112.6 ± 0.5	115.7 ± 0.5
		(112.5; 114.3)	(111.7; 113.5)	(111.7; 113.5)	(114.7; 116.6)
	75–100%	111.6 ± 0.7^*^	113.2 ± 0.7	113.8 ± 0.7	120.0 ± 0.7^*^
		(110.3; 112.9)	(111.9; 114.5)	(112.5; 115.1)	(118.6; 121.4)
Men	0–25%	102.8 ± 0.2	103.1 ± 0.2	103.4 ± 0.2	104.2 ± 0.2^*^
		(102.5; 103.2)	(102.7; 103.5)	(103.0; 103.8)	(103.8; 104.6)
	25–50%	102.2 ± 0.2	102.4 ± 0.2	102.6 ± 0.2	102.4 ± 0.2
		(102.0; 102.5)	(102.1.5; 102.6)	(102.3; 102.8)	(102.1; 102.6)
	50–75%	100.2 ± 0.3*^$^^£^*	100.0 ± 0.3^*^	101.4 ± 0.3^$^	102.6 ± 0.3
		(99.7; 100.8)	(99.5; 100.5)	(100.9; 101.9)	(102.0; 103.1)
	75–100%	100.8 ± 0.*^$^^£^*	101.7 ± 0.4^$^	105.6 ± 0.4^*^	106.3 ± 0.4^*^
		(100.0; 101.5)	(101.0; 102.4)	(104.9; 106.3)	(105.6; 107.0)

*Mean lap time (expressed in s) ± SD and 95% confidence intervals in parentheses. The race was divided in distinct race parts: 0–25, 25–50, 50–75, and 75–100% with 0% being the beginning and 100% being the end of the race. Riders are sorted as a function of the final position with Q1, Q2, Q3, and Q4 being the first, second, third and last quartile*.

* *Significant difference with the other parts of the race*.

$ *significant difference with 0–25%*.

£ *Significant difference with 25–50%. P <0.05 was used as the level of statistical significance. For simplicity differences between quartiles are not shown (see Results section)*.

### Positioning

In contrast to lap time, riders' positioning demonstrated huge variability (lap-by-lap position is shown as supplementary figures for women and men, Supplementary Figures 1, 2). Mean coefficients of variation were 51.6% for women and 54.3% for men. The individual differences between the best and worst position during the race ranged between 3–31 for women and 6–52 for men.

**Figure 3:**
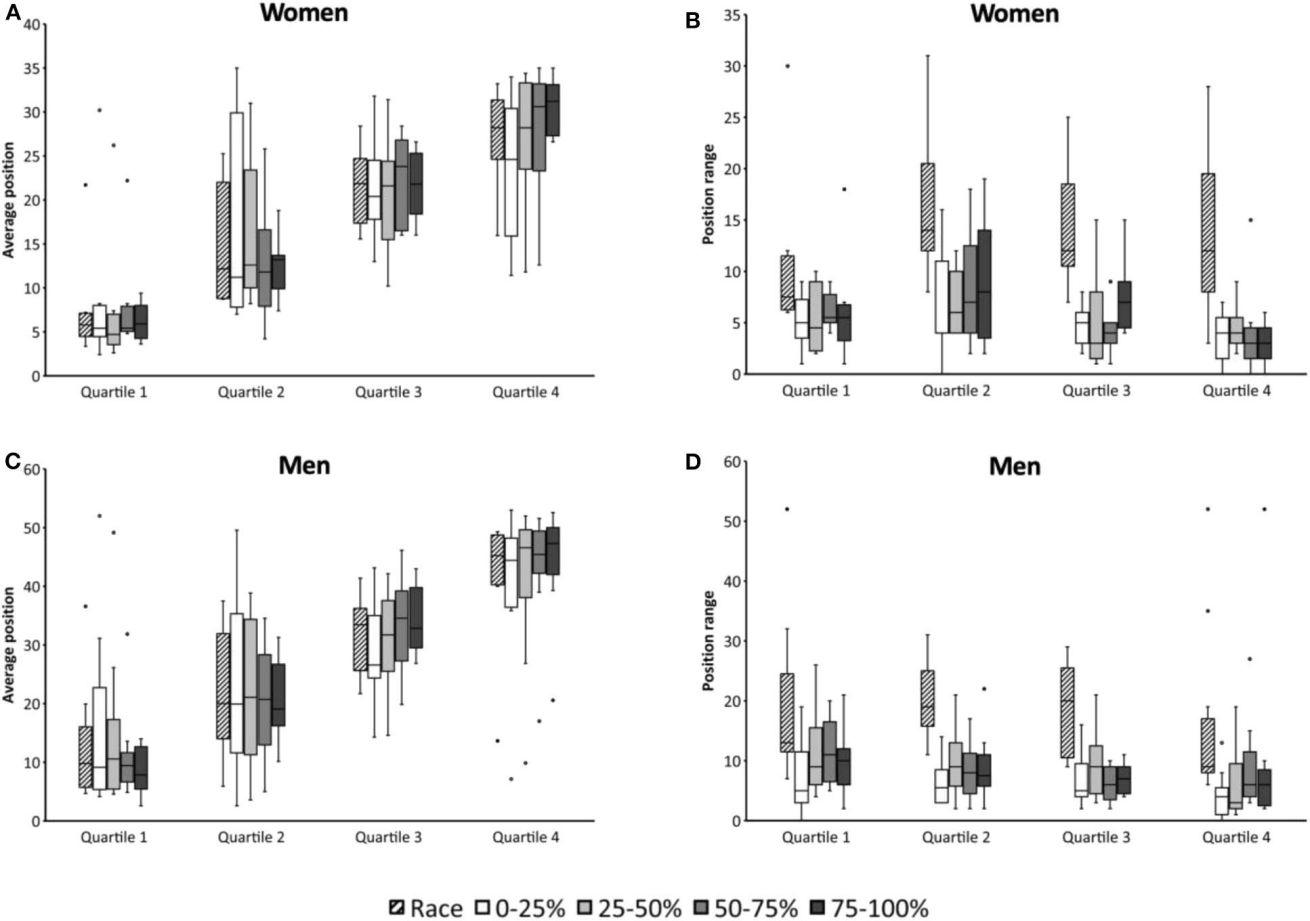
Whisker plots representing riders' position difference between the race mean position **(A,C)** and the final position **(B,D)** for women **(A,B)** and men **(C,D)**. Positive values correspond to position gains and negative values to position losses. Dots represent outliers. Riders are sorted as a function of the final position with Q1 being the faster riders.

The mean riders' position and positioning range during the whole race and within the race are presented as whisker plots in Figure 3. When considering the whole race and the different parts (0–25, 25–50, 50–75, and 75–100%), no difference was observed between the final position and the mean position (*P* > 0.05). This finding is confirmed by the correlations (*P* < 0.05) obtained between the final position and the mean riders' position when considering the race as a whole or in distinct parts (Table 2).

**Table 2:** Correlation with the final position for mean position and position range during the race.

	**Mean position**		**Position range**	
	**Women**	**Men**	**Women**	**Men**
Whole race	0.664^*^	0.689^*^	−0.150	0.175
0–25%	0.434^*^	0.475^*^	−0.067	−0.239^*^
25–50%	0.562^*^	0.557^*^	−0.385^*^	−0.232^*^
50–75%	0.629^*^	0.720^*^	−0.268^*^	−0.235^*^
75–100%	0.863^*^	0.832^*^	0.086	−0.100

*The whole race is presented, or it was divided in distinct parts: 0–25, 25–50, 50–75, and 75–100%*.

* *significant correlation (P <0.05)*.

When considering quartiles, the mean position for Q1 was lower than Q3 and Q4 for women and men (*P* < 0.05). Q2 had a lower mean position than Q4 during the whole race for men and during the second half of the race for women (*P* < 0.05). Figure 3 also demonstrated a large positioning dispersion. Some outliers were present in Q1 for both women and men and Q4 for men. A lower dispersion was observed for Q1 in women as compared to Q2, Q3, and Q4. In contrast, the dispersion was almost similar for men whatever the quartile, but it was reduced during the second half of the race for Q1.

When considering position range, large variations were obtained. Position changes when considering lap-by-lap ranged from [−19; +16] and [−39; +20] for women and men, respectively (positive values corresponding to position gains). Because huge variations are obtained, lap-by-lap was not considered for analysis (only race parts, Figure 3). Position range appeared negatively correlated with respect to the final position for 50–75 and 75–100% for women and for 25–50, 50–75, and 75–100% for men (Table 2). The best the riders are, the greatest the position variation is. For women, statistical analyses revealed that during 50–75 and 75–100%, Q2 had greater position range than Q3 and Q4 (*P* < 0.05). Within a given quartile, position range was similar when considering the different parts of the race. For men, Q1 had greater position range than Q3 during 50–75% (*P* < 0.05). For Q2, position range was smaller during 0–25% than 25–50% (*P* < 0.05). For Q4, position range was smaller during 0–25% than 75–100% (*P* < 0.05). No difference between quartile was obtained when considering the entire race for position range.

## Discussion

The aim of the present study was to explore pacing and positioning during a top-level fixed-gear criterium. By nature, fixed-gear cycling during a criterium imposes a variable pacing strategy within a single lap as a result of racing in a short closed-circuit with lots of turns and therefore numerous accelerations and decelerations. Despite small lap time variations, the main results of our study demonstrated a variable pacing strategy during the race. It has large physiological and tactical consequences. The first consequence is to lower positioning variation during the second half of the race. From positioning analyses, it clearly appeared that some riders used a drafting strategy that could be detrimental in case of insufficient technical skills.

Considering lap time, very small variations were observed as attested by very low coefficients of variation. Such values (<5%) have already been observed (Billat et al., 2001) in track runs at free pace. However, beside small variability, significant accelerations, and decelerations were observed within the race in women and men that could have large physiological and tactical consequences. The faster laps were observed at the middle and at the end of the race. They are generally preceded and followed by slow laps. Such race behavior is not surprising and is generally referred as variable pacing (fluctuation in intensity during exercises) (Liedl et al., 1999; Abbiss and Laursen, 2008). Generally, this pacing strategy is used and recommended to counteract variable racing conditions such as geography or wind (Swain, 1997; Atkinson et al., 2007; Cangley et al., 2011). However, it has previously been suggested to maintain this pacing strategy within a small range (<5% of the mean power output) to be tolerated by cyclists (Faria et al., 2005). Moreover, some authors suggested that variable pacing strategies were less efficient than even-paced strategies (constant pace, Thomas et al., 2012).

With variable pacing, greater physiological responses (e.g., blood lactate) and perception of efforts have been observed (Palmer et al., 1997; Thomas et al., 2012). As a result of accelerations/decelerations, most authors argue that the variable pacing strategy resulted in excessive glycogen depletion (mostly on type II fibers) and premature onset of fatigue (Palmer et al., 1999; Faria et al., 2005). Accordingly, at mid-race during the present study, fast laps may have large impacts on the final results and on the rider's positioning. It would permit to discriminate fast from slow riders both in women and men races. Indeed, accelerations will exacerbate fatigue in slow riders who will be unable to follow the race head during the subsequent laps. Consequently, women from the last quartile had slower lap times during the second half of the race. For men, riders from the second half were slower than the first half during the second part of the race. As obtained here, increases of the exercise intensity at the end of the race are commonly observed. It is suggested to result from increases in motor unit recruitment and use of anaerobic energy reserve (Faria et al., 2005). Practically, it permitted to substantially discriminate riders and to get favorable positioning for the ultimate sprint. Because of the trace of the race it necessitated huge attentional, technical, and tactical skills. A low fatigue level that is more likely obtained at race end for the best riders might favor these specific skills (Lorist et al., 2002).

While lap time and the associated variable pacing strategy is mostly associated with fatigue and riders' selection, analyzing positioning give additional important information. Riders' position revealed very large variability (with coefficients of variation >50%). This variability is related to the rules of such events that favor grouped races and therefore numerous positioning variations. As previously indicated, lap-by-lap position gains could be >15 positions. Data analyses from the present study indicated that positioning might have important impact on the final results and is partly a consequence of lap time. Whatever the race part, the mean rider's position has been shown to be significantly correlated with the final position. Obviously, the correlation increased from the beginning to the end of the race. Whisker plots appear appropriate to observe such interesting behaviors. Riders from Q1 are mostly amongst the first during the whole race. Nevertheless, the dispersion in averaged position is more reduced in women than men. It could indicate that finalists were more heterogeneous in the women race than men. Except for a single outlier, women from Q1 are always within the 10 first riders.

Whisker plots also appear appropriate to identify some outliers. From Q1, a single rider in the women as well as in the men race demonstrate being mostly positioned at the rear of the race. It denoted a clear drafting tactic, well-know and often used in various sports such as cycling, running, speedskating or even horse racing (Krieg et al., 2006; Spence et al., 2012; Heimans et al., 2017). Drafting clearly reduces air resistance, energy utilization and therefore fatigue (Faria et al., 2005). However, these single cases are only extreme behaviors of the general strategy used by riders because it has large hazard. For example, a race accident is easily observed in Q4 with one outlier that was in the first positions during the whole race and finished in the last position only because of the ultimate and fastest lap. When considering more carefully data, this drafting strategy is not so exceptional. While only few riders from Q1 have such strategy, it appears that riders from Q2 in the women and men race have large mean position dispersions mostly during the first half of the race. In contrast, riders from Q3 and Q4 mostly rode at the rear of the race.

As previously indicated, positioning appeared to be a consequence of lap time. Such conclusion is clearly visualized in Figures 3A,C with a reduced dispersion of riders' averaged position particularly during the second half of the race (coinciding with the first acceleration). Interestingly, position variations (as attested by position range) were not correlated or even negatively correlated with the final position. A negative correlation was mostly observed in the middle part of the race and revealed that the larger the position variation is, the better the riders are. However, such finding should be cautiously considered since riders from the rear of the race have inherently reduced variations within the last positions of the race. Accordingly analyses by quartiles are more appropriate. Moreover, statistics on position range only revealed some marginal differences. Accordingly, riders from the different quartiles behave almost similarly.

Finding the appropriate position during drafting is challenging for riders. Indeed, taking benefits of drafting might reduce fatigue as a result of aerodynamics drag but might contrarily increase fatigue as a result of increased cognitive processes while directing the bicycle to avoid contacts with other riders. It is well-known that prolonged cognitive activity would negatively impact endurance performance and that saving mental efforts could benefit to physical efforts maintenance (Radel et al., 2017; Van Cutsem et al., 2017). To reduce this potential extra fatigue, riders should therefore be familiar with such technical skills. It should be remembered that the difficulty of such skills is reinforced by the particularity of fixed-gear bicycles (continuous pedaling and brakeless).

## Conclusion

Our results revealed a similar variable pacing strategy in women and men during an international fixed-gear criterium. Accelerations were observed at mid-race and at the end of the race. They have large impact in riders positioning. These accelerations have large impact in riders positioning. Analyzing riders positioning indicated that the best riders mostly rode within the first positions (i.e., fast start). These riders started fast and could impose speed variations within the race. However, drafting could appear an interesting strategy during the first half of the race to slow fatigue development and therefore to be able to accelerate at mid-race. However, technical skills while directing the bicycle are also important factors during training not to develop detrimental extra-fatigue. In addition to the aerobic system, previously shown as being an important performance determinant, practitioners should be specifically trained for these multiple accelerations and decelerations in some particular race parts. Moreover, some technical skills facilitating the ability to ride grouped and to navigate inside a group should be specifically trained to reduce the potential extra-fatigue induced by the attentional cognitive processes.

## Data Availability Statement

Publicly available datasets were analyzed in this study. This data can be found here: https://data.mendeley.com/datasets/thnfscmhnf/2.

## Ethics Statement

Ethical review and approval was not required for the study on human participants in accordance with the local legislation and institutional requirements. Written informed consent for participation was not required for this study in accordance with the national legislation and the institutional requirements.

## Author Contributions

NB, CC, and CP designed the study. MT and DT collected all data. NB performed statistical analyses and wrote the first draft of the paper. All authors revised and approved the submission.

## Conflict of Interest

MT and DT were employed by Trimble racing Inc. The remaining authors declare that the research was conducted in the absence of any commercial or financial relationships that could be construed as a potential conflict of interest.
