# Supplementary material for: Pacing and Positioning Strategies During an Elite Fixed-Gear Cycling Criterium
Source: Front Sports Act Living. 2020 Oct 8;2:586568. doi: 10.3389/fspor.2020.586568 (PMC7739637; doi:10.3389/fspor.2020.586568)
Supplement: Supplementary file 1 [file Data_Sheet_1.docx]

**Figure S1.** Lap-by-lap position for women. Final position is presented in x-axis (#1 being the winner). Lap is presented in y-axis. The different colors represent the position range. For example, 0-10 indicates riders are within the 10 first riders of the race for the given lap.


**Figure S2.** Lap-by-lap position for men. Final position is presented in x-axis (#1 being the winner). Lap is presented in y-axis. The different colors represent the position range. For example, 0-20 indicates riders are within the 20 first riders of the race for the given lap.
